# Change in frailty status in the 12 months following solid organ transplantation: a systematic review and meta-analysis

**DOI:** 10.1093/ageing/afae283

**Published:** 2025-01-08

**Authors:** Anna Bevan, Jenny Avery, Hoe Leong Cheah, Ben Carter, Jonathan Hewitt

**Affiliations:** Centre for Medical Education, Heath Park, Cardiff University, CF14 4YS; Centre for Medical Education, Heath Park, Cardiff University, CF14 4YS; Centre for Medical Education, Heath Park, Cardiff University, CF14 4YS; Department of Biostatistics and Health Informatics, Institute of Psychiatry, Psychology and Neuroscience, King’s College London, De Crespigny Park, SE5 8AF, London; Department of Population Medicine, Heath Park, Cardiff University, CF14 4YS

**Keywords:** frailty, surgery, solid organ transplant, systematic review, older people

## Abstract

**Objectives:**

To investigate if frailty status alters following solid organ transplantation (lung, liver, kidney and heart) without rehabilitation intervention.

**Research design and methods:**

Studies published between 1 January 2000 and 30 May 2023 were searched across five databases. Studies measuring frailty, using a validated or established frailty measure, pre- and post-transplant were included. Narrative synthesis was used to describe the included studies according to the time post-transplant and according to solid organ group. Where data allowed a meta-analysis was conducted to compare frailty prevalence pre- and 6–12 months post-transplant across studies.

**Results:**

Twelve studies were included in this review (6 kidney transplant, 2 liver transplant, 3 lung transplant and 1 heart transplant), with a total of 3065 transplant recipients with 62% being male. The mean age across studies was 51.35 years old. When narratively synthesised after an initial worsening of frailty immediately post-transplant, there appears to be a significant improvement in frailty by 3 months post-transplant that is sustained by 6 to 12 months following solid organ transplantation. Five studies were included in the meta-analysis which demonstrated an odds ratio = 0.27 (95% CI, 0.12, 0.59, *P* = .001, ${I}^2$ = 82%) for frailty prevalence post-solid organ transplantation (SOT) compared to frailty prevalence pre-SOT. When the single paper deemed to be of poor quality was removed the remaining four studies demonstrated a reduced odds ratio of being frail at 6–12 months post-transplant (OR 0.45 (95% CI, 0.32, 0.65, *P* = .001, ${I}^2$ = 13%).

**Conclusions:**

Transplant may be associated with a reversal in frailty, although heterogeneity was demonstrated across studies.

## Key Points

- Frailty appears to be reversible following solid organ transplant.
- This systematic review includes heart, liver and kidney transplant.
- The effect was consistent for each organ studied.

## Introduction

Solid organ transplantation (SOT) has transformed the survival and quality of life of people living with advanced organ disease (AOD), offering a life-saving treatment for diseases considered otherwise terminal [1]. In recent decades, advances in surgical techniques and immunosuppressive therapy have made SOT accessible to older and more complex patients, thus making frailty pertinent to consider in SOT [1].

Frailty is characterised by reduced physiological reserves and failure of homeostatic mechanisms. This results in an increased vulnerability to adverse health outcomes following minor stressor events [2–4]. Frailty is prevalent in AOD and has been found to develop at a younger age in patients with AOD when compared with the general population [5–8]. As advances in SOT have made possible SOT in an older patient, demand for SOT will increase [9]. With a limited amount of donor organs available, there is a need to prioritise patients by their likelihood to benefit from SOT, including by their pre-transplantation frailty status [9]. Although a limited number of transplant criteria (e.g. Model for End-Stage Liver Disease) have now become standard in certain SOTs, patients are mainly prioritised for SOT via subjective clinical opinion [9]. Development of a more objective, holistic and comprehensive frailty assessment for all patients awaiting a SOT will be more desirable moving forwards.

Despite this, growing evidence supports positive post-transplant outcomes [4]. Multiple studies across all SOT groups have evidenced improvements in health related quality of life (HRQOL) and key factors of frailty including physical function, sarcopenia and physiological reserves [10–12]. Of significant interest amongst these studies are that positive impacts are seen across age groups, and despite significant pre-transplant frailty [13]. Furthermore, since the suggestion by Flint and colleagues (2012) that frailty may be reversible with the insertion of a left ventricular assist device (LVAD) in cardiac failure patients, a growing body of literature has begun to explore the potential of SOT to reverse frailty [14–18]. However, no study has explored the reversibility of frailty, in the absence of rehabilitation intervention, following SOT across all transplant groups. This systematic review aims to establish if SOT is associated with a reduction of frailty (heart, liver, lung and kidney).

## Methods

### Study design

This systematic review and meta-analysis were conducted within the Preferred Reporting Items for Systematic reviews and Meta-Analyses (PRISMA) framework (Supplemental File 1). The review was registered, and the protocol made available in the International prospective register of systematic reviews (PROSPERO; https://www.crd.york.ac.uk/prospero/). Registration number CRD42023399018.

### Search strategy

The search strategy was developed in collaboration with an expert research support librarian at the University of Cardiff. Two authors (AB and JA) independently searched five electronic databases including MEDLINE, CINAHL, EMBASE, The Cochrane library and Web of Science. The search was conducted in May 2023. The search terms were based on Medical Subject Headings (MeSH) terms and other controlled vocabulary. Any disputes were mediated by a third author (JH). The detailed search strategy is outlined in Supplemental File 2. Identified and relevant studies’ references were manually reviewed to identify any potential studies that met the inclusion criteria. The included studies underwent a forward citation search.

### Study selection: inclusion and exclusion criteria

Studies were included if an objective frailty status measurement was used pre- and post-SOT, and SOT was performed during the study. Studies were also screened to ensure that no rehabilitation took place pre- or post-transplant. This ensures that any noted reduction in frailty status can be associated with SOT alone.

All experimental and observational cohort studies tracking patient frailty status pre- and post-SOT, available in English, published between 1 January 2000 and 30 May 2023 were included. Studies were not selected before the year 2000 to ensure the surgical and medical management of these patients remained up to date.

Studies were excluded if they did not include a validated objective measurement of frailty status, if there were no pre- and post-SOT measurement of frailty status, or if patients in the studies took part in rehabilitation pre- or post-SOT. Two studies were also excluded on the basis that there was no access to the main texts of these studies.

### Primary outcome of frailty

Studies were included if they measured frailty using any validated instrument pre- and post-SOT at any time. The following were examples of those included: fried frailty phenotype (FFP) [3], clinical frailty scale (CFS) [19] Groningen frailty indicator (GFI) [20] and Short Physical Performance Battery (SPPB) [21]. Also included were studies using reputable frailty measures specific to SOT organ groups, as recommended within a recent expert consensus statement in frailty in SOT [4] Examples of those included were: Modified FFP [3, 22] and Liver Frailty Index (LFI) [18]. The frailty instruments used are summarised in Supplemental File 4.

### Data extraction and quality assessment

Data were manually extracted for included studies and collated into a pre-assembled table. Some pre frailty data were presented for prevalence but for the subsequent analysis pre- and frailty participants were grouped together.

The Newcastle Ottowa Scale (NOS) was used to assess the risk of bias in the included studies. It considers three domains: selection, comparability and exposure. Each domain is scored and determined as good, fair or poor. Under the domain of selection, the studies were considered ‘good’ if they score at least 3 points, ‘fair’ if they scored 2 points and ‘poor’ if they scored 1 or 0 points. Under the domain of selection, studies may be awarded a point each if: (i) the study population selected is truly representative of the average patient population undergoing organ transplant, (ii) the study population underwent SOT with no pre- or post-transplant rehabilitation, (iii) baseline measurement of frailty pre-transplant using a validated objective measure of frailty status was described, or (iv) the study population not undergoing solid organ transplant, if there was any, had similar baseline characteristics to the study population SOT. Under the domains of comparability and exposure respectively, the studies were considered ‘good’ if they score at least 2 points, ‘fair’ if they scored 1 point and ‘poor’ if they scored 0 points. Under the domain of comparability, studies may be awarded a point each if: (i) there was adequate adjustment for pre-existing frailty in the study population, or (ii) adequate adjustment for confounding factors influencing the outcome of transplant surgery. Under the domain of comparability, studies may be awarded a point each if: (i) post-transplant frailty was measured using a validated objective measure of frailty status, (ii) study participants post-transplant were accounted for including participants lost to follow-up, or (iii) there was sufficient time allowed after transplant to assess for post-transplant frailty. Studies were deemed good quality if they scored good in all domains, fair if they scored fair in one or more domain and poor if they scored poor in any one domain. Please refer to Supplemental File 7 for results of the quality assessment process.

### Data synthesis

Where studies were available with clear baseline characteristics of study participants and data was available at both baseline and post-baseline outcome time-point they were considered for pooling into a meta-analysis. Data extracted at 6 and 12 months post-transplant data was pooled in a random-effects meta-analysis using the Mantel Haenszel method. The random-effects model was selected due to the expected high heterogeneity in the pooled data [23]. Meta-analysis data were presented as odds ratio (OR) with the associated 95% confidence intervals (95%CI), p-values, and ${I}^2$ measure of heterogeneity. For studies with data at 6 and 12 months, the data at 12 months was used. Studies were collated using Review Manager 5.1 (RevMan 5) software.

### Assessment of subgroups and statistical heterogeneity

Statistical heterogeneity was assessed using the ${I}^2$ statistic. Heterogeneity exceeding 80% was explored using subgroup analysis [23]. Subgroups were pre-determined including organ group, frailty measure used and quality assessment (fair and moderate vs poor quality).

Additionally, studies were narratively described per organ group to consider characteristics of included studies and frailty changes over time per organ group. Factors reported to influence frailty changes were also considered within each organ group.

## Results

### Identified studies and quality assessment

**Figure 1:**
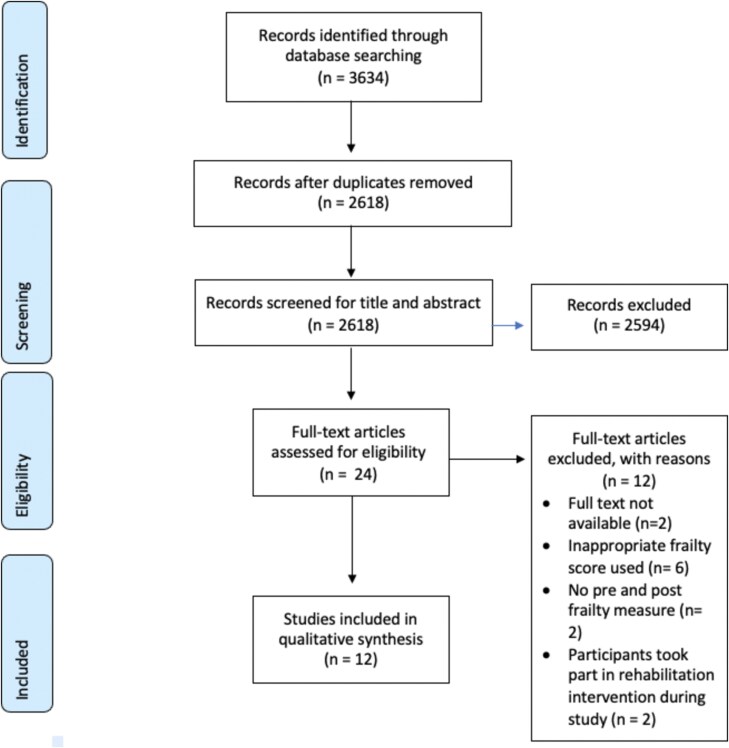
PRISMA Flow Diagram of included studies.

After removal of duplicates 2618 records were identified. 24 full texts were reviewed, leading to 12 being excluded (see Supplemental File 6 for titles and reasons for exclusion).Twelve studies were eventually included within this systematic review as shown in the PRISMA flowchart (Figure 1).

### Characteristics of the included studies

The included studies were published between 2015 and 2023, and of the 12, all were observational studies (Supplemental File 3). We have not specifically excluded any other study design. The 12 studies included a total of 3065 SOT recipients, and 62% were men. The mean age across studies was 51.35 years old. SOT groups represented were kidney transplant (KidneyTx; *n* = 6), liver transplant (*n* = 2), lung transplant (LungTx; *n* = 3) and heart transplant (HeartTx; *n* = 1). All 12 studies compare frailty pre- and post-transplantation using an appropriate measure of frailty. Although included studies did not specifically state that patients did not receive rehabilitation, all included studies with the exception of Perez *et al.* 2020 [24] measured pre-transplant frailty status only in the immediate period leading up to transplant (only up to a week pre-surgery), and post-transplant frailty were mostly measured during routine outpatient clinics. Quint *et al.* 2020 [25], in particular, measured post-transplant frailty via telephone contact with participants. Therefore, based on these data we have extrapolated that these studies did not intend to include rehabilitation as part of their assessment of pre- and post-transplant frailty.

### Quality assessment of included studies

Quality assessment resulted in 6 studies being deemed good quality [14, 18, 26–29], 4 fair quality [15, 16, 24, 25, 30, 31] and 2 of poor quality [16, 31] (Supplemental File 7). No studies were excluded based on quality assessment however this was considered by subgroup analysis within the meta-analysis.

### Frailty prevalence of included studies

Of the included studies, 5 studies presented data on frailty prevalence before, and 6 to 12 months after SOT. Two in KidneyTx recipients [15, 31], Two in liverTx [18, 26] and one in LungTx [29] (Supplemental File 5).

Of these only Aroca-Martinez and colleagues [31] and Venado and colleagues [29] presented comprehensive follow up data. With Venado, demonstrating a higher death rate in people living with frailty post-transplant, with a 6 month Hazard Ratio 2.28 (1.07–4.88; Supplemental File 5).

### Short term change in frailty (<6 months post-transplant)

6 studies report on changes in frailty within the first 6 months following SOT representing kidney, liver and lung transplant recipients [18, 24, 27–30]. Overall, most studies (4/6) report an improvement in frailty status within the first 6 months following SOT.

Contrastingly, one study by Lai *et al.* report a significant increase in frailty amongst liver transplant recipients measured using the LFI 3 months post-transplant [18].

This overall improvement in frailty appears to follow an initial increase in frailty early post-transplant reported in LungTx and KidneyTx recipients [27, 32]. Notably, the one study included in HeartTx patients did not report on frailty in HeartTx recipients within the first 6 months post-transplant.

### Summary

Eight studies report frailty status at 6 to 12 months post-SOT (Jha *et al.* 2017; Lai *et al.* 2018; Perez *et al.* 2020; Venado *et al.* 2019; Quint *et al.* 2020; Lai *et al.* 2022; Aroca-Martinez *et al.* 2023; [15, 16, 18, 24–26, 31]). Lai *et al.* [18] and Venado *et al.* [29] report frailty at 6 and 12 months post-transplant. These studies represent all SOT groups: 2 studies in liver-Tx [18, 26, 29], 2 in LungTx [24, 33], 3 in KidneyTx [15, 25, 31] and 1 in HeartTx recipients [16].

### Frailty status 6 months post-transplant

At 6 months 4 studies report a statistically significant improvement in frailty status (Venado *et al.* 2019; Aroca-Martinez *et al.* 2023; Jha *et al.* 2017; Perez *et al.* 2020). Aroca-Martinez *et al.* (2023), measure frailty using the CFS in 57 KidneyTx recipients all of whom received haemodialysis prior to transplant. They report a significant improvement in CFS (from 4 (vulnerable) to 3 (robust), *P* < .01) after 6 months of KidneyTx. Similarly, Jha *et al.* [16] report that frailty scores improve significantly amongst 13 HeartTx recipients with frailty measures pre- and post-transplant. However, Lai *et al.* [18] report no significant change in frailty 6 months following liver-Tx.

### Frailty status 12 months post-transplant

Amongst liver transplant recipients significant improvements in frailty are reported by 12 months post-transplant when measured using the LFI [18, 34]. Contrastingly one study in KidneyTx recipients by Quint and colleagues [25] report that frailty increases 12 months post-transplant, attributed to non-frail recipients becoming frail when measured by the Groningen Frailty Index (GFI).

**Figure 2:**
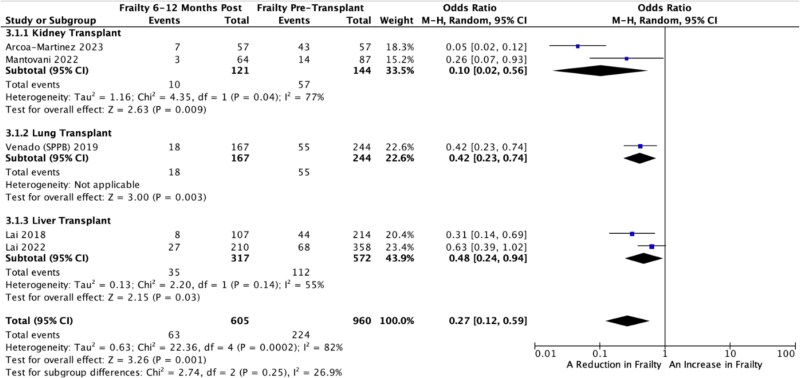
Forest-plot of frailty prevalence at 6 to 12 months post-SOT.

Venado *et al.* [29] measured frailty by SPPB and FFP; however, the number of people who underwent assessment by each frailty measure was not mutually exclusive; therefore, only those measured by SPPB (*n* = 244/246) were included in the pooled meta-analysis. We compared the number of people frail before SOT with the number of people frail post-SOT within a meta-analysis and there was a substantial reduction in frailty in those who underwent transplant, compared to those that did not. The pooled OR = 0.27 (95% CI, 0.12, 0.59, *P* = .001, ${I}^2$= 82%), the heterogeneity was partially explained by subgrouping the studies in the transplanted organ (Figure 2).

### Long term changes in frailty (12 months post-transplant or longer)

Three of the included studies present long-term data, beyond 12 months post-transplant [14, 25, 29]. Venado *et al.* [29], report a plateau in frailty improvements at 6 to 36 months post-SOT, following the initial significant improvement within the early period (up to 6 months). Quint *et al.* [25], report that after a mean follow-up period of 22.8 ± 8.3 months frailty prevalence increased from 17% to 26.7%. This increase in frailty prevalence is detected by a self-reported measure of frailty, the GFI.

### Subgroup analysis of poor versus fair and good quality studies

**Figure 3:**
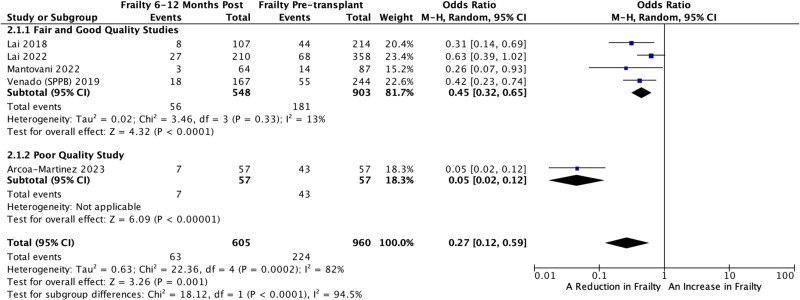
A subgroup analysis of poor vs fair and good quality studies.

Only one of the studies included in the meta-analysis was deemed poor quality [31]. When this study was removed from the analysis the OR for the reduction in frailty status was 0.45 (95% CI, 0.32, 0.65, P = 0.001, ${I}^2$= 13%); see Figure 3.

### Medium term changes in frailty (6 to 12 months post-transplant)

## Discussion

Twelve studies were identified that assessed frailty on transplant patients, and most were of good or fair quality. In the medium term a reduction in associated frailty status is noted across SOT groups, with frailty prevalence reducing at 6 to 12 months post-transplant.

Whilst this describes the overall trend in frailty across organ groups it is important to acknowledge variations in specific organ groups and large heterogeneity across the studies. We also note a mean age of transplant recipients across all studies of 51.35 years. As such, the reduction in frailty status measurement noted across these groups of patients may not be replicable across an older patient group.

Potential mechanisms for reversing frailty following SOT have been proposed. It is possible that restoring organ function may aid a reversal of key influences of frailty, including micronutrient deficiencies, cachexia, physical inactivity, sarcopenia and even chronic inflammation following SOT transplantation [4, 35]. There have also been associations with improved quality of life following SOT [4, 32]. For instance, patients who had a heart transplant in treatment for severe heart failure have noted a significant quality of life improvement [32, 36]. In particular, Wilhelm and colleagues note reduced symptom burden in these patients (exercise tolerance is much improved and patients are able to resume normal activities of daily living without feeling out of breath) [36]. This in turn led to better functional quality of life as some patients were able to return to work at least on a part time basis. It is noted that improvements in quality of life and symptom burden is not a direct measurement of frailty and may represent an improvement in other aspects of patient care, such as a reduction of fatigue seen in end stage organ failure. However, the noted association between a reduction in frailty status and SOT would account for at least part of the quality of life improvements noted in previous studies.

However, this review identifies frailty following SOT as a highly dynamic process, with recipients transitioning from frail to non-frail and non-frail to frail states across organ groups, mirroring findings in community dwelling older adults and those awaiting SOT [5, 8, 37].

Furthermore, numerous studies reported no significant influence of age on frailty trajectories following SOT [14, 29], supporting the suggestion that age should not be a contraindication to transplantation, but rather that work should be done to distinguish between frailty resulting from chronological age, that is unlikely to improve following transplantation, and frailty attributed to the failure of the organ with the potential to reverse [4].

It is important to note that within the study by Jha *et al.* [16] included in this review, data surrounding non-frail recipients who may have transitioned to a state of increased frailty following HeartTx are not provided, possibly leading to an over exaggeration of the effect. Furthermore, given the suggestion that following LVAD there may be changes in frailty at different points in time, the significant variation in the time (184 [88–457] days) of frailty reassessments alongside the very small sample limit the generalisability of this result. Flint and colleagues also suggest that older patients with multiple co-morbidities and advanced heart disease are less likely to demonstrate reversibility of frailty after major surgical interventions [38]. Therefore, whilst these findings following LVAD and HeartTx are promising, further large scale longitudinal research, across age groups, is required to determine if frailty is reversible at different points in time following HeartTx.

There were limited studies included within this review exploring frailty immediately post-SOT and beyond 12 months post-SOT, and therefore the findings of this review in relation to these time periods should be treated with caution. Beyond 12 months post-SOT findings within this review are limited to 3 studies [14, 25, 29].

### Strengths and limitations

A range of frailty measures were used, and frailty assessments were conducted at different times pre- and post-transplant frailty assessments, making drawing comparisons much more difficult and preventing pooling of many data. Further, the degree of improvement in frailty is difficult to ascertain, particularly as data were grouped for the analysis into pre- and frail participants. Finally, the included studies had a high drop-out rate, with a high percentage lost to follow-up. Whilst this is representative of the nature of post-transplant follow-up, multiple studies failed to account for these participants or draw comparisons with those remaining. It therefore remains a possibility that many of the frail participants died and this bias may account for some or all of the reduction in frailty that was recorded in these studies. Along with substantial heterogeneity, meaning the findings should be interpreted with caution.

All the included studies used validated or well-established measures of frailty measured pre- and post-SOT. Further the majority of the studies were of good or fair quality. When the single poor quality study, that was included in the meta-analysis was removed, the OR for the improved changed from 0.27 to 0.45 but the heterogeneity was much lower at *I*^2^ = 13%.

## Conclusion

This review concludes that a reduction in frailty status may be associated with SOT across organ groups, especially in the medium term. However, we identify frailty post-SOT as a highly dynamic process, whereby frail recipients become non-frail, whilst non-frail recipients pre-transplant may also transition to states of increased frailty.

## Supplementary Material

aa-23-1980-File002_afae283
