# Supplementary material for: Change in frailty status in the 12 months following solid organ transplantation: a systematic review and meta-analysis
Source: Age Ageing. 2025 Jan 8;54(1):afae283. doi: 10.1093/ageing/afae283 (PMC11962592; doi:10.1093/ageing/afae283)
Supplement: aa-23-1980-File002_afae283 [file aa-23-1980-file002_afae283.docx]

**Change in Frailty Status in the 12 Months Following Solid Organ Transplantation: A Systematic Review and Meta-analysis**

Supplementary Files

## Supplemental File 1: PRISMA 2020 checklist for reporting systematic reviews.

| **Section and Topic** | **Item #** | **Checklist item** | **Location where item is reported** |
| --- | --- | --- | --- |
| **TITLE** | | |  |
| Title | 1 | Identify the report as a systematic review. | Title |
| **ABSTRACT** | | |  |
| Abstract | 2 | See the PRISMA 2020 for Abstracts checklist. | Abstract |
| **INTRODUCTION** | | |  |
| Rationale | 3 | Describe the rationale for the review in the context of existing knowledge. | Introduction, paragraph 3 |
| Objectives | 4 | Provide an explicit statement of the objective(s) or question(s) the review addresses. | Introduction, paragraph 4 |
| **METHODS** | | |  |
| Eligibility criteria | 5 | Specify the inclusion and exclusion criteria for the review and how studies were grouped for the syntheses. | Study Selection; Supplemental Files 6, 7, and 8 |
| Information sources | 6 | Specify all databases, registers, websites, organisations, reference lists and other sources searched or consulted to identify studies. Specify the date when each source was last searched or consulted. | Search Strategy; Supplemental File 2 |
| Search strategy | 7 | Present the full search strategies for all databases, registers, and websites, including any filters and limits used. | Supplemental File 2 |
| Selection process | 8 | Specify the methods used to decide whether a study met the inclusion criteria of the review, including how many reviewers screened each record and each report retrieved, whether they worked independently, and if applicable, details of automation tools used in the process. | Search Strategy |
| Data collection process | 9 | Specify the methods used to collect data from reports, including how many reviewers collected data from each report, whether they worked independently, any processes for obtaining or confirming data from study investigators, and if applicable, details of automation tools used in the process. | Data extraction and Quality Assessment |
| Data items | 10a | List and define all outcomes for which data were sought. Specify whether all results that were compatible with each outcome domain in each study were sought (e.g., for all measures, time points, analyses), and if not, the methods used to decide which results to collect. | Data synthesis |
|  | 10b | List and define all other variables for which data were sought (e.g., participant and intervention characteristics, funding sources). Describe any assumptions made about any missing or unclear information. | Data synthesis |
| Study risk of bias assessment | 11 | Specify the methods used to assess risk of bias in the included studies, including details of the tool(s) used, how many reviewers assessed each study and whether they worked independently, and if applicable, details of automation tools used in the process. | Data extraction and Quality Assessment |
| Effect measures | 12 | Specify for each outcome the effect measure(s) (e.g., risk ratio, mean difference) used in the synthesis or presentation of results. | Data synthesis |
| Synthesis methods | 13a | Describe the processes used to decide which studies were eligible for each synthesis (e.g., tabulating the study intervention characteristics and comparing against the planned groups for each synthesis (item #5)). | Identified Studies and Quality Assessment |
|  | 13b | Describe any methods required to prepare the data for presentation or synthesis, such as handling of missing summary statistics, or data conversions. | Assessment of Subgroups and Statistical Heterogeneity |
|  | 13c | Describe any methods used to tabulate or visually display results of individual studies and syntheses. | Supplemental File 3 |
|  | 13d | Describe any methods used to synthesize results and provide a rationale for the choice(s). If meta-analysis was performed, describe the model(s), method(s) to identify the presence and extent of statistical heterogeneity, and software package(s) used. | Assessment of Subgroups and Statistical Heterogeneity |
|  | 13e | Describe any methods used to explore possible causes of heterogeneity among study results (e.g., subgroup analysis, meta-regression). | Assessment of Subgroups and Statistical Heterogeneity |
|  | 13f | Describe any sensitivity analyses conducted to assess robustness of the synthesized results. | Assessment of Subgroups and Statistical Heterogeneity |
| Reporting bias assessment | 14 | Describe any methods used to assess risk of bias due to missing results in a synthesis (arising from reporting biases). | Data extraction and Quality Assessment |
| Certainty assessment | 15 | Describe any methods used to assess certainty (or confidence) in the body of evidence for an outcome. | Data synthesis |
| **RESULTS** | | |  |
| Study selection | 16a | Describe the results of the search and selection process, from the number of records identified in the search to the number of studies included in the review, ideally using a flow diagram. | Identified Studies and Quality Assessment |
|  | 16b | Cite studies that might appear to meet the inclusion criteria, but which were excluded, and explain why they were excluded. | Supplemental File 6 |
| Study characteristics | 17 | Cite each included study and present its characteristics. | Supplemental File 3 |
| Risk of bias in studies | 18 | Present assessments of risk of bias for each included study. | Supplemental File 7 |
| Results of individual studies | 19 | For all outcomes, present, for each study: (a) summary statistics for each group (where appropriate) and (b) an effect estimates and its precision (e.g., confidence/credible interval), ideally using structured tables or plots. | Supplemental File 3 |
| Results of syntheses | 20a | For each synthesis, briefly summarise the characteristics and risk of bias among contributing studies. | Supplemental File 7 |
|  | 20b | Present results of all statistical syntheses conducted. If meta-analysis was done, present for each the summary estimate and its precision (e.g., confidence/credible interval) and measures of statistical heterogeneity. If comparing groups, describe the direction of the effect. | Changes to frailty at 6 months posttransplant; Changes in frailty status at 6 to 12 months posttransplant; Changes in frailty status beyond 12 months posttransplant |
|  | 20c | Present results of all investigations of possible causes of heterogeneity among study results. | Changes to frailty at 6 months posttransplant; Changes in frailty status at 6 to 12 months posttransplant; Changes in frailty status beyond 12 months posttransplant |
|  | 20d | Present results of all sensitivity analyses conducted to assess the robustness of the synthesized results. | Changes to frailty at 6 months posttransplant; Changes in frailty status at 6 to 12 months posttransplant; Changes in frailty status beyond 12 months posttransplant |
| Reporting biases | 21 | Present assessments of risk of bias due to missing results (arising from reporting biases) for each synthesis assessed. | Changes to frailty at 6 months posttransplant; Changes in frailty status at 6 to 12 months posttransplant; Changes in frailty status beyond 12 months posttransplant |
| Certainty of evidence | 22 | Present assessments of certainty (or confidence) in the body of evidence for each outcome assessed. | Changes to frailty at 6 months posttransplant; Changes in frailty status at 6 to 12 months posttransplant; Changes in frailty status beyond 12 months posttransplant |
| **DISCUSSION** | | |  |
| Discussion | 23a | Provide a general interpretation of the results in the context of other evidence. | Discussion, paragraph 1 |
|  | 23b | Discuss any limitations of the evidence included in the review. | Discussion, paragraphs 3, 4, and 5 |
|  | 23c | Discuss any limitations of the review processes used. | Strengths and limitations |
|  | 23d | Discuss implications of the results for practice, policy, and future research. | Conclusion |
| **OTHER INFORMATION** | | |  |
| Registration and protocol | 24a | Provide registration information for the review, including register name and registration number, or state that the review was not registered. | Prospero |
|  | 24b | Indicate where the review protocol can be accessed, or state that a protocol was not prepared. | Prospero |
|  | 24c | Describe and explain any amendments to information provided at registration or in the protocol. | No amendments |
| Support | 25 | Describe sources of financial or non-financial support for the review, and the role of the funders or sponsors in the review. | No source of financial support |
| Competing interests | 26 | Declare any competing interests of review authors. | No competing interests |
| Availability of data, code, and other materials | 27 | Report which of the following are publicly available and where they can be found template data collection forms; data extracted from included studies; data used for all analyses; analytic code; any other materials used in the review. | Supplemental File 3 |

## Supplemental File 2: Detailed search strategy

Embase Classic+Embase <1947 to 2023 Week 09>

Ovid MEDLINE(R) ALL <1946 to March 03, 2023>

Search Strategy:

1 exp Frailty/ (33676)

2 Frail*.mp. (99450)

3 (muscle adj1 wast*).mp. (15449)

4 exp Sarcopenia/ (28332)

5 sarcopenia.mp. (42267)

6 exp Muscular Atrophy/ (81417)

7 exp Lung Transplantation/ (60846)

8 exp Liver Transplantation/ (202903)

9 exp Kidney Transplantation/ (291876)

10 Heart Transplantation/ (99828)

11 lung transplant*.mp. (77034)

12 liver transplant*.mp. (233130)

13 kidney transplant*.mp. (289882)

14 heart transplant*.mp. (123312)

15 (major organ adj1 transplant*).mp. (71)

16 7 or 8 or 9 or 10 or 11 or 12 or 13 or 14 or 15 (705682)

17 1 or 2 or 3 or 4 or 5 or 6 (191893)

18 16 and 17 (3659)

19 limit 18 to English language (3605)

20 limit 19 to yr="2000 -Current" (3537)

## Supplemental File 3: Table of included studies

| **Author; Year.**  **Study Design** | **No. Patients**  **% pre-frail/ frail pre and post SOT** | **Age**  ± = SD  () = range  **Gender**  %Male | **Description of Population** | **Frailty Tool** | **Time of Pre Measure** | **Time of Post Measure** | **Pre Frailty Score** | **Post Frailty Score** | **Key Findings** |
| --- | --- | --- | --- | --- | --- | --- | --- | --- | --- |
| **Kidney Transplant Studies** | | | | | | | | | |
| Mantovani 2022  Prospective single-centre cohort study | 64  15.6%  4.7% | 44.9±12.2  64% Male | Patients with end-stage kidney disease listed for KidneyTx | FFP | At admission for surgery | 12 months  (11-13 months) | 1 | 1 | No significant change in median frailty score but percentage reduction in frailty was significant – patients gained weight in the first year posttransplant  Participants lost to follow up were older. |
| Lorenz  2017  Prospective single-centre cohort study | 140  NA | 51.2±15.1  61.4% Male | Adult living donor KidneyTx recipients | SPPB | Within 1 week of transplant | 4 months  (33-196 days) | 11.11 | 11.43 | Means of all measures of physical performance improved significantly.  Decreased physical function associated with longer LOS and rehospitalisation  Low pre-tx physical function reported worse QOL after transplant – association for significant |
| Quint  2020  Prospective single-centre cohort study | 176  17%  32%  27%  22% | 51.8±14.1  63.1% Male | Living and diseased donor KidneyTx recipients. | GFI | On admission for surgery | 1,2 and 3 years  (22.8±8.3 months) | NA | NA | Almost one-fifth of non-frail transitioned to frail after transplant.  Cognitive impairment strong indicator of frailty.  Non-frail who transitioned to frail were older - not significant.  No significant associations were found in risk of transitioning to another frailty state. |
| Aroca- Martinez 2023  Prospective Cohort study | 57  75%  12% | 42±13  (20-71)  63% Male | Adult KidneyTx recipients who had been on haemodialysis for >3 months | Clinical Frailty Scale  (CFS) | Immediately before surgery | 6 months | 4 | 3 | CFS improved from 4 to 3, a significant improvement.  Mildly frail to mildly robust.  After 6 months 97% improved their frailty score. 11% remained the same and 2% worsened. |
| Chu  2022  Prospective 2-centre cohort study | 1336  16%  NA | 52.7±14  60.3% Male | Adult KidneyTx recipients | FFP | On admission for surgery | 1,3,6,12 up to 5 years | NA | NA | Lower likelihood of frailty over time in the first 2.5 years post-KidneyTx (aOR = 0.96, p< .001)  But higher likelihood of frailty over time between 2.5 and 5 years (aOR = 1.03, p= .03).  Frail patients were older and more likely to have cognitive impairment but had similar post trajectories.  Older patients (≥65) 1.73 fold more likely to have frailty at time of admission than younger (18-64) |
| McAdams-DeMarco 2015  Prospective single-centre cohort study | 349  19.8%  33.3%  27.7%  17.2% | 53.3±4.2  (19-83)  61.9% Male | Adult KidneyTx recipients who had more than one post-operative follow-up | FFP | On admission for surgery | 1,2 and 3 months  Mean 14 m | NA | NA | Frailty initially worsens but then improves by 3 months.  Those frail at KidneyTx had higher frailty scores in the long term.  Pre-transplant frailty is not an irreversible state and changes are similar in older adults. |
| Liver Transplant Studies | | | | | | | | | |
| Lai  2018  Prospective single-centre cohort study | 214  21%  21%  10%  7% | 62  (56-66)  64% Male | Liver transplant recipients with cirrhosis who had at least 90 days of follow-up posttransplant | Liver Frailty Index  (LFI) | 2.4 (1.2-4.4) months | 3,6 and 12 months  23.2(15.4-30.0) | 3.7 | 3.9 – 3.7 -3.4 | Frailty worsens by 3 months and improves modestly by 12 months. Fewer than 2 of 5 achieved robustness. |
| Lai  2022  Prospective multi-centre cohort study | 358  19%  13% | 60  IQR 53-65  68% Male | Liver transplant recipients with cirrhosis | LFI | At each pre visit, variable timing  Frail 1.7 m  Non 2.8 m | 12 months | 3.9 | 3.4 | Pre-frailty associated with worse functional health 1 year post.  LFI scores posttransplant significantly worse in those with pretransplant frailty. Frailty associated with worse HRQOL in physical domains. Frailty improved overall. |
| Lung Transplant Studies | | | | | | | | | |
| Venado  2019  Prospective single-centre cohort study | 246  SPPB 23%  FFP 43%  6 months  SPPB 7%  FFP 11% | 59  IQR 49-66  56% Male | Adults undergoing first time lung transplant 72% restrictive lung disease 15% obstructive | SPPB and FFP | SPPB  76 (0-441) days  FFP  95 (0-769) days | 3,6,12,18,24,30 and 36 months  Median 2.4 (1.0-3.4) years | SPPB  Frail 5.1  Non frail 10.5  FFP  Frail 3.2  Non frail 1.4 | NA | Frailty defined by SPPB and FFP improved to a clinically meaningful extent early after transplant (0-6 months) and remained stable thereafter.  Approx. 84% of survivors who were frail before transplant became non frail after. |
| Perez  2020  Prospective single-centre cohort study | 23  22%  Unclear | 31±7.9  48% Male | Adult lung transplant recipients with cystic fibrosis in ‘Breath Again’ study | SPPB | Every 3 months on waiting list | 3 and 6 months | 9.8 | 11.4 – 10.8 | Clinically significant reduction in frailty by 6 months posttransplant. Of 5 subjects frail pretransplant, four were not frail 6 months after. |
| Mayer  2021  Retrospective single-centre cohort study | 25  0%  NA | 57±13  68% Male | Adult end-stage lung failure patients who underwent lung transplant | SPPB | 3 days building up to surgery | At hospital discharge  23 (18-40) days | 11.5 | 7.7 | SPPB score got worse posttransplant. |
| Heart Transplant Studies | | | | | | | | | |
| Jha  2017  Prospective single-centre cohort study | 100  (77 HeartTx)  23%  0% | 51±13  (16-71)  65% Male | Patient who underwent major surgical intervention for advanced heart failure | Modified- Fried Frailty Phenotype  (mFFP) | Immediately before surgery | 184 days (88-457) | 3.3 | 0.8 | Significant improvement with all frail patients to non-frail post-HeartTx. Followed only those who were frail pretransplant.  Only 13 HeartTx recipients underwent post-assessment |

CI = confidence interval MCID = Minimal Clinical Importance Variable

## Supplemental File 4: Frailty assessment tools used in included studies

| Reference | Frailty Scale | Items Measured | Scoring | Administration |
| --- | --- | --- | --- | --- |
| Fried et al 2001b | Fried Frailty Phenotype  (Physical Frailty Phenotype) | 5 domains:  Slowness  Physical activity  Weight Loss  Exhaustion  Weakness | Score range: 0 to 5  Frail ≥ 3  Pre-frail = 1-2  Non-frail = 0 | Physician and self-reported |
| Fried et al 2001a  Romero-Ortuno et al 2010 | Modified Fried Frailty Phenotype (mFFP) | 5 domains:  Exhaustion  Grip-strength  Mobility  Appetite  Physical activity | Score range: 0 to 5  Frail ≥ 3  Non frail < 3 | Physician and self-reported |
| Rockwood et al 2005 | Clinical Frailty Scale (CFS) | 9 categories:   1. Very fit (robust, active) 2. Well (no active disease but less fit then “very fit”) 3. Managing well (medial problems are well controlled, people not regularly active) 4. Vulnerable (not dependent but symptoms limit activities) 5. Mildly frail (limited dependence in high order IADLs) 6. Moderately frail (help needed with outside activities, with keeping house, with bathing) 7. Severely frail (completely dependent but stable) 8. Very severely frail (completely dependent, approaching the end of life) 9. Terminally ill (approaching the end of life, with life expectancy < 6 months) | A physician assigns a score based on an assessment and review of medical records.  Frail ≥ 5 | Physician administered |
| Steverink et al 2001 | Groningen Frailty Indicator (GFI) Questionnaire | 15-item questionnaire  Physical components:  Are you able to carry out these tasks single-handedly and without any help?  9 questions follow  Cognitive components:  Do you have complaints about your memory?  Social components:  Do you sometimes experience emptiness around yourself?  Do you sometimes miss people around yourself?  Do you sometimes feel abandoned>?  Psychological component:  Have you recently felt downhearted or sad?  Have you recently felt nervous or anxious? | Eight items have a two response category (yes/no), six items have three response category (yes/sometimes/no), and one item has a Likert response (1-10)  A higher score indicates greater level of frailty, with a maximum score of 15.  Frail ≥ 4 | Self-reported |
| Lai et al 2017 | Liver Frailty Index (LFI) | 3 performance based measures  Grip strength  Timed chair stands  Balance testing | 3-performance based tests of physical function are input into a calculator available at:  <http://liverfrailtyindex.ucsf.edu>  Frail ≥ 4.5  Pre-frail 3.2 – 4.4  Robust < 3.2 | Objective measures completed by physician |
| Guralnik et al 2000 | Short Physical Performance Battery (SPPB) | Group of measures combining results of 3 factors:  Gait speed  Chair stand  Balance tests | Score range: 0-12  0 = worst performance  12 = best performance  Frail ≤ 7  Non-Frail > 7 | Physician administered |

## Supplemental File 5: Studies reporting frailty prevalence pre-transplant and 6-12 months posttransplant.

| **Studies** | **Number and Percentage of People Frail Pre-transplant** | **Number and Percentage of People Frail 6 to 12 Months Posttransplant.** | **Follow up data presented** |
| --- | --- | --- | --- |
| **Aroca-Martinez 2023** | 43/57 (75%) | 7/57 (12%) | No loss to follow up |
| **Lai 2016** | 44/214 (21%) | 8/107 (7%) | 46 died, no analyses by frailty status |
| **Lai 2022** | 68/358 (19%) | 27/210 (13%) | No mortality data presented |
| **Dos Santos Mantovani 2022** | 14/87 (16%) | 3/64 (5%) | 46 died, no analyses by frailty status |
| **Venado 2019 (SPPB)** | 55/244 (23%) | 18/167 (11%) | 46 died, with a higher rate of death in people living with frailty |

## Supplemental File 6: Table of Excluded Studies

| **Author** | **Title** | **Reason for Exclusion** |
| --- | --- | --- |
| Courtwright 2018 | Cause, preventability, and cost of unplanned rehospitalisation’s within 30 days of discharge after lung transplantation | Modified SPPB not validated |
| Courtwright 2019 | Discharge frailty following lung transplantation | Mention of rehabilitation unclear if participants undertook prehabilitation before transplant therefore influencing results. |
| Jha 2016 | Reversibility frailty advanced heart failure patients listed for transplantation | Abstract only |
| McDonald  2019 | Reversibility of frailty in LVAD and heart transplant patients | Abstract only |
| Montgomery  2020 | Reversibility of frailty after lung transplant | Patients completed a detailed rehabilitation program before posttransplant frailty measure. |
| Rozenberg 2018 | Frailty and clinical benefits with lung transplantation | Does not give pre and post frailty measure using validated/ appropriate frailty measure |
| Venado  2020 | Frailty after lung transplantation is associated with impaired HRQOL and mortality | Does not give pre and post SPPB value. |
| Singer  2017 | Effect of Lung Transplantation on Health-Related Quality of Life in the Era of the Lung Allocation Score: A U.S. Prospective Cohort Study. | No validated objective measure of frailty |
| Ali H.  2021 | Impact of kidney transplantation on functional status | KPS not a validated frailty measure/ measure of physical function only |
| Thuluvath  2018 | Karnofsky performance status before and after liver transplantation predicts graft and patient survival | KPS not a validated frailty measure/ measure of physical function only |
| Michelson 2018 | Association between TUGT at transplant evaluation outcome after kidney transplantation | TUGT not validated frailty measure |
| McAdams-DeMarco 2018 | Frailty and post kidney transplant health related QOL | Fried Frailty Phenotype measures pre-transplant however then only used to compare HRQOL following – no re-measure of frailty posttransplant in isolation. |

## Supplemental File 7: Quality Assessment Studies.

| Study Author | Selection^&^ | | | | Comparability* | | Outcome* | | | Quality Assessment  ** | Comments |
| --- | --- | --- | --- | --- | --- | --- | --- | --- | --- | --- | --- |
|  | 1 | 2 | 3 | 4 | 1 | 2 | 1 | 2 | 3 |  |  |
| Mantovani 2022 | 0 | 1 | 1 | 1 | 1 | 0 | 1 | 1 | 0 | Fair | Lost to follow-up were older. No adjustment for confounding variables/ comparability between frail and non-frail. Small sample size does not reach power calculation. |
| Lorenz 2017 | 0 | 1 | 1 | 1 | 1 | 0 | 1 | 1 | 1 | Fair | Multiple measures of performance used. Single centre. Excluded deceased donor. Single-centre small sample exclusion of diseased donors. |
| Quint 2020 | 0 | 1 | 1 | 1 | 1 | 1 | 0 | 1 | 0 | Fair | Self-reported frailty measure taken by telephone – GFI validated tool. Limited description of selection process. Excluded those deceased from previous cohort. |
| Aroca-Martinez 2023 | 0 | 1 | 0 | 1 | 0 | 0 | 0 | 1 | 0 | Poor | Inaccurate reporting of study design. Only those on dialysis, small sample from a single centre. No limitations reported. |
| Chu 2022 | 1 | 1 | 1 | 1 | 1 | 1 | 1 | 1 | 1 | Good | Long follow-up bias due to attrition (sensitivity analysis). Large sample. |
| McAdams-DeMarco 2015 | 1 | 1 | 1 | 1 | 1 | 1 | 1 | 1 | 0 | Good | Limited follow up time. % followed up low at each time point. |
| Lai 2018 | 1 | 1 | 1 | 1 | 1 | 1 | 1 | 1 | 0 | Good | Lost to follow up 50% by 12 months – no description/ comparison of lost. |
| Lai 2022 | 1 | 1 | 1 | 1 | 1 | 1 | 1 | 1 | 1 | Good | FrAILT study only in ambulatory setting – cannot be applied to inpatient |
| Venado 2019 | 1 | 1 | 1 | 1 | 1 | 1 | 1 | 1 | 0 | Good | High drop out by 12 and 36 months from relatively small sample. Mostly restrictive lung disease. |
| Perez 2019 | 0 | 1 | 1 | 1 | 1 | 0 | 1 | 1 | 1 | Fair | Only CF patients. Small sample only 23 patients. |
| Mayer 2021 | 0 | 1 | 1 | 1 | 1 | 1 | 1 | 0 | 1 | Good | Very small sample 25 patients. Short follow-up period not likely to see changes in frailty. |
| Jha 2017 | 0 | 1 | 0 | 1 | 1 | 0 | 1 | 1 | 0 | Poor | Small sample size – lost to follow-up on 13/77 underwent frailty reassessment. Limited description. |

Domain scored^&^: Good (3+); fair (2); poor (0-1).

Domain scored*:Good (2-3); fair (1); poor (0)

**For a study to be classed as good quality, it had to score ‘good’ for every domain, 2 domains were deemed as fair quality and one or no domains as poor quality
